# Out-of-Pocket Expenditure and Challenges Faced by Patients Undergoing Heart Valve Replacement in Follow-Up Care at a Tertiary Care Center in South India: A Mixed Methods Study

**DOI:** 10.7759/cureus.66127

**Published:** 2024-08-04

**Authors:** Athul M Remesh, Arivarasan Barathi, Arunkumar Ravichandran, Mahalakshmy Thulasingam, Hemachandren Munusamy

**Affiliations:** 1 Obstetrics and Gynaecology, Jawaharlal Institute of Postgraduate Medical Education and Research, Puducherry, IND; 2 Community Medicine, Employee's State Insurance Corporation (ESIC) Medical College and Hospital, Chennai, IND; 3 Clinical Research, Jawaharlal Institute of Postgraduate Medical Education and Research, Puducherry, IND; 4 Preventive and Social Medicine, Jawaharlal Institute of Postgraduate Medical Education and Research, Puducherry, IND; 5 Cardiovascular Surgery, Jawaharlal Institute of Postgraduate Medical Education and Research, Puducherry, IND

**Keywords:** oral anticoagulant therapy (oat), rheumatic heart disease, prosthetic heart valve, out of pocket expenditure, inr monitoring

## Abstract

Background

Heart valve replacement surgery is one of the most commonly performed cardiac surgeries in India. Post-surgery, the patient requires lifetime anticoagulation therapy with regular follow-up, leading to financial and nonfinancial burdens for the patients. This study aimed to determine the out-of-pocket (OOP) expenditure (OOPE) for follow-up visits to the heart valve clinic and explore and assess the challenges faced by patients during these follow-ups.

Methodology

This mixed methods study was conducted at a tertiary care center from June 2018 to August 2018, focusing on patients attending the Valve Replacement clinic. The qualitative component of the study involved conducting three focus group discussions, which were transcribed and manually analyzed using thematic analysis to generate categories. The monthly OOPE and the proportion of irregular patients were assessed using a pretested and validated questionnaire developed based on the findings from the qualitative study. The data from the quantitative study were entered into EpiData version 3.1 (EpiData, Odense, Denmark) and analyzed using Stata 14 (StataCorp., College Station, TX).

Results

The median (interquartile range [IQR]) total OOPE for patients was Rs. 765 (475-1,100). The median (IQR) direct and indirect expenditures were Rs. 420 (210-600) and Rs. 590 (330-948), respectively. The patients faced difficulties in the categories of financial, travel, hospital, family, and personal. Out of a total of 143 participants, 86 (60.14%) had incurred catastrophic health expenditures. The cost also significantly increased with the presence of an accompanying person and longer travel durations.

Conclusions

The major difficulties faced by the patients were distance and expense. Telemedicine can help overcome these challenges by decentralizing follow-up care to the primary care level.

## Introduction

Valvular heart disease is one of the most prevalent cardiac surgical conditions causing morbidity and mortality in India [1]. Consequently, valve replacement surgery is one of the most commonly performed cardiac surgery [2]. In patients who undergo valve replacement surgery, the newly placed mechanical valve possesses a high risk of thrombosis [3]. This mandates the initiation of lifelong anticoagulation in such patients [4]. Since anticoagulation possesses a high risk of bleeding and a high degree of food and drug interactions, regular and periodic monitoring of such patients is important to titrate the drug levels to achieve targeted anticoagulation and to avoid any therapy-related adverse events and impose a burden of life long monthly visits to the hospitals [5,6].

However, in a public health care set up in a country like India, these major surgeries are being undertaken in tertiary care referral centers located in cities wherein the majority of the patients present to the OPD from far and remote areas [7]. This poses huge difficulties and inconveniences to the patients for long-term follow-up at these faraway centers [8]. Since the follow-up of the majority of patients post valve replacement is solely for the objective of monitoring the laboratory value of the international normalized ratio (INR) [9], ideas for alternate and simpler methods to follow up the patients at nearby centers would be of prime importance in such tertiary health care setups. This leads to poor compliance among the patients with irregular follow-up. Since the difficulty faced by the patients. can adversely affect the frequency of follow-up visits, easing the process can help improve the same [10].

There is a dearth of published literature identifying the difficulties faced and expenditure incurred during the monthly follow-up of these valve replacement patients visiting referral centers and their impact on the frequency of follow-up visits. Thus quantifying the difficulties faced and losses by such patients would prove helpful in exploring the need for alternate methods of follow-up like telemedicine for such patients from remote areas especially in poor resource settings [11]. Among heart valve replacement patients attending the Heart Valve Clinic at a tertiary care referral center in South India, this study aimed to determine the out-of-pocket (OOP) expenditure (OOPE) for follow-up visits, explore and assess the difficulties faced by patients during these visits, and determine the proportion of patients who missed the recommended follow-up appointments.

## Materials and methods

The study employed a mixed methods design, categorized as exploratory, with a qualitative study followed by a quantitative study. It was conducted at a weekly Heart Valve Clinic of a tertiary care referral center in South India, held every Wednesday. The clinic serves all valve replacement follow-up patients from the hospital. As an institute of national importance, the hospital caters to the resident population of Puducherry, nearby Tamil Nadu, and other states. Approximately 15 valve replacements are performed each month at the institute, and around 120 patients attend each Heart Valve Clinic for follow-up. Thus, approximately 500 valve replacement patients are under regular follow-up each month at this clinic. The study was conducted over two months, from June 2018 to August 2018, after obtaining clearance from the Institutional Ethics Committee (JIP/2018/0143).

For OOPE, the sample size was calculated using the formula (Z*S.D/E)^2, where *Z* is 1.96 for a 95% confidence interval, SD is the standard deviation, and E is the precision. With the expected mean (SD) of OOPE as Rs. 1952.06 (1208.12) and a precision of 200 units, the sample size was calculated as 140.

Inclusion criteria

Patients who underwent heart valve replacement at least 12 months before the study and were on monthly follow-up at the Heart Valve Clinic of the tertiary care center during the study period were included.

Exclusion criteria

Patients who underwent combined procedures, such as coronary artery bypass graft with valve replacement and patients who experienced heart failure or any other complications, such as prosthetic valve obstruction, after valve replacement surgery were excluded.

Procedure

The study was conducted at the Heart Valve Clinic of a tertiary care referral center after obtaining clearance from the Institutional Ethics Committee, JIPMER. The qualitative study included three focus group discussions (FGDs) with four to five willing participants meeting the inclusion criteria. Participants were selected using purposive sampling, which could introduce selection bias. To minimize selection bias, efforts were made to include diverse demographics and ensure representation from various socioeconomic backgrounds. Before the discussions, an open-ended semi-structured guide was developed based on initial literature and insights from the clinical team. Key areas of focus included cost, travel difficulties, and emotional burdens experienced during follow-ups. Each session was held in a private setting within the outpatient clinic to ensure confidentiality and minimize distractions. The moderators introduced the topics and guided the discussion using prompting questions, allowing for organic conversation that encouraged participants to freely express their thoughts and experiences. The sessions were recorded (with consent) and transcribed verbatim for analysis. Participants were encouraged to engage in free-flowing discussion. The discussions aimed to identify common expenses and difficulties faced by the patients. The FGDs were audio-recorded, and transcripts were created. Based on the FGD findings, a questionnaire was developed for the quantitative study. Common problems and economic losses incurred were documented. 

In the quantitative study, both the expenditure and the problems faced by patients were quantified. The proportion of patients with irregular follow-ups was also determined. A draft questionnaire was initially prepared after reviewing the literature and was later modified based on the results of the qualitative study. It was pre-tested with three patients. All patients were interviewed at the clinic after obtaining informed consent, and data were collected. They were asked about the ease, time, and mode of travel to the clinic, as well as direct and indirect expenditures, duration of stay at the hospital, and any other economic and non-economic losses faced. Additionally, they were asked for their opinions on alternative follow-up methods, such as telemedicine.

Operational definitions

OOPE refers to the direct expense borne by the valve replacement patient for a single follow-up visit that is not recovered or reimbursed. 

Direct expenses will include medical and nonmedical expenses. The medical expenses include consultation charges, laboratory charges, and medicine charges. The nonmedical direct expenses include the transport charges, cost of food and accommodation, and expenses of the accompanying person. 

Indirect costs include tangible and intangible expenses. Tangible cost includes the loss of wages during the visit.

Follow-Up Visits

The institute recommends at least one follow-up visit every month. Any number of visits fewer than this is considered inadequate.

Data Analysis

Qualitative data: Audio-recorded FGDs were transcribed verbatim within a week. Manual content analysis was done by two independent researchers to generate thematic categories. It was reviewed by another person to strengthen interpretive credibility.

Quantitative data: Quantitative data were entered in EpiData version 3.1 and analyzed using Stata 14. The cost was summarized as the median and interquartile range (IQR). The costs incurred were compared across various sociodemographic/clinical characteristics using the Kruskal-Wallis test. A *P*-value less than 0.05 was considered statistically significant.

## Results

A total of 143 participants were included in the study, of whom 68 (47.55%) were male. The sociodemographic data of the participants are depicted in Table 1. The median (IQR) age of the participants was 39 (31-47) years. Most participants (53.85%) had pursued secondary schooling. The majority of participants were from areas outside Puducherry (93.01%), and their median (IQR) one-way travel time for each follow-up visit was 3 (2-5) hours. The majority of participants (70.63%) had been on follow-up for about one to five years. The median (IQR) monthly income of the study participants was Rs. 6,000 (4,000-10,000) (Table 1).

**Table 1:** Out-of-pocket costs based on sociodemographic details of the study participants on monthly follow-up at the Heart Valve Replacement Clinic in a tertiary care hospital (N = 143). Kruskal-Wallis (*P *> 0.05, significant).

Variable	Frequency	Total cost (rupees), median (IQR)	Direct cost (rupees), median (IQR)	Indirect cost (rupees), median (IQR)
Total	143	765 (475-1,100)	600 (300-850)	200 (0-400)
Age in completed years				
10-35	53 (37.06)	855 (550-1,185)	690 (500-900)	0 (0-300)
>35	90 (62.94)	700 (400-1,100)	553 (280-800)	50 (0-350)
Sex				
Males	68 (47.55)	900 (500-1,200)	600 (300-850)	300 (0-400)
Females	75 (52.45)	665 (417-1,020)	600 (340-800)	0 (0-51)
Educational status				
Educated	118 (82.5)	780 (500-1,100)	600 (300-850)	1 (0-300)
Uneducated	25 (17.5)	735 (370-1,125)	600 (310-860)	0 (0-300)
Time of travel				
≤3.5	83 (58.0)	698 (383-1,030)	575 (260-780)	100 (0-300)
>3.5	60 (42.0)	900 (510-1,190)	680 (450-954)	0 (0-300)
Accompanying person				
Yes	50 (35.0)	835 (600-1,350)*	700 (570-1,000)*	0 (0-300)
No	94 (65.0)	698 (375-1,035)	550 (270-800)	200 (0-300)

All patients who attended follow-up incurred out-of-pocket expenditures (OOPEs). The median OOPE among the subjects was Rs. 600, with a minimum of Rs. 50 and a maximum of Rs. 2,500. The median (IQR) OOPE for participants was Rs. 765 (475-1,100). The median (IQR) direct and indirect costs were Rs. 600 (300-850) and Rs. 200 (0-400), respectively. Total OOPE was higher among younger age groups compared to older age groups, although this difference was not statistically significant. It was higher for males (Rs. 900) than for females (Rs. 717) and increased with longer hours of travel. The cost also significantly rose in the presence of an accompanying person (Table 2).

**Table 2:** Out-of-pocket expenditure of the participants (heart valve replacement patients) for one monthly follow-up visit to a Heart Valve Clinic at a tertiary care center in Puducherry, India. *Median (IQR) of the observation whose values > 0. IQR, interquartile range

Cost (N = 143)	Median (IQR)
Total expenditure	765 (475-1,100)
Out-of-pocket expenditure	600 (300-850)
Direct medical	0 (0-400)
Hospital expenditure	0 (0-0)
Medications	0 (0-400)
Direct nonmedical	335 (196-600)
Transport	250 (100-460)
Food	100 (60-200)
Accommodation	0 (0-0)
Indirect	0 (0-300)
Indirect tangible* (*n *= 50)	300 (250-400)

Out of a total of 143 participants, 86 (60.14%) incurred catastrophic health expenditure (CHE) due to monthly follow-up visits to a tertiary care center in Puducherry, India, where CHE was defined as total OOPE exceeding 10% of monthly family income [12].

About 30% of the participants were irregular in attending the monthly follow-up visits. It was found that the regularity of follow-up among the study participants was associated with the period after surgery, which was statistically significant with a *P*-value of 0.013, which was analyzed by chi-square test. The regularity was also associated with the time travel and expenses incurred. We also found that the percentage of participants with difficulties related to distance, mode of travel, expense, and work were 37.8, 28.0, 32.9, and 30.8, respectively. However, they were statistically insignificant with a *P*-value > 0.05. Additionally, out of the total participants surveyed, 73% were willing to use telemedicine for follow-up at nearby centers. 

Regarding qualitative analysis, patients faced difficulties in five main categories: financial, travel, hospital-related, family, and personal. Participants experienced financial difficulties such as insufficient funds, increased bus fares, and medication costs, which often forced them to take out loans and created a financial burden. They also lost wages for a day or more due to follow-up visits, further exacerbating their financial situation (Table 3).

**Table 3:** Summary of qualitative data analysis of the participant’s opinion on the difficulties faced and suggestions on monthly follow-up visits following heart valve replacement in a tertiary care center in Puducherry, India. PT-INR, prothrombin time-international normalized ratio

Theme 1: Challenges faced	
Category 1: Financial	
Inadequate money	A 48-year-old male says, "If we do not have money, we even come the next week. Then we even buy the medicines for that week at the medical shop."
Forced to take loans	A 54-year-old male says, "We will have to take loans and repay on getting salary."
Loss of wages on follow-up days	Two participants with a job say, "We might not be able to go to work on the next day even due to tiredness and all. Also we might be losing wages even for the accompanying person."
Forced to compromise on basic needs such as food	A 20-year-old female housewife says, "My father doesn’t have food the whole day. He only eats after going home in the night worrying what if there is no money at home or what if there is no money for bus. He buys for me and gives me."
Increased bus charges	A 48-year-old female says, "They have increased the bus fares too."
Increased cost of medications	A 30-year-old female says, "They have increased the cost of tablets to be bought outside."
Increase in expenditure due to an accompanying person	A 48-year-old female says, "We have to come all alone for the checkup. Otherwise it will increase the expenses and bus fare also has gone up now."
Category 2: Travel	
Health-related issues due to long travel	A 40-year-old male from Dindigul says, "I get back pain travelling in the bus."
Long-duration travel	Mrs. Rani says, "It is also very much tiring to travel this long."
Unavailability of buses	A 38-year-old male from Dindigul says, "I have to leave the previous day itself, at about 8 in the evening." A 40-year-old female from Neyveli says, "There are no much buses from our place."
Unavailability of a direct transport facility to the hospital	A 40-year-old male says, "Bus is there but you have to board multiple connecting buses."
Category 3: Healthcare facility	
Repeat visits in the same month due to deranged PT-INR value	A 38-year-old female says, “It becomes more difficult when there is a problem in the test result. We will have to come back again after a day or two then.”
Occasional unavailability of laboratory services	A 38-year-old female says “Once there was no kit available for PTINR and had to check it outside.”
Inability to access the pharmacy facility	A 54-year-old male says, “We are sometimes buying the medicines outside if we come for follow up a bit late[Since they might miss the bus to their place due to the long queues at the hospital pharmacy].”
Category 4: Family and social causes	
Difficulty to leave the elderly alone	A 30-year-old female says, “It’s also difficult to leave old aged people alone at home.”
Has to depend on others to care for people at home	A 48-year-old man says, “My daughter has to take leave when I come for follow up to take care of parents at home.” A 48-year-old male says, “In my house there no one to take care. My father in law who is about 20kms far comes to take care of my parents.”
Miss out on child education needs	A 38-year-old female says, “Sometimes I’m also not able to attend the parents meeting at my children’s school due to these follow-up visits.”
Accompanying person’s employment issues	A 28-year-old female says, “If he[Brother, the accompanying person] comes[for follow-up] his job for a day and others’ job for whom he provides job too will go.”
Theme 2: Willingness to take up telemedicine follow-up	
Willing to take up telemedicine	A 48-year-old female from Panruti says, “Yes, Why not…we are ready to take it up.”
Concerns about the medications	A 40-year-old female from Thirukkovilor says, “Yes[In response to a Question whether she is willing to take up Telemedicine follow up],but sometimes when we take medicines from local hospitals, when we bring it here they say it’s the wrong medicine.”
Not willing to take up telemedicine	A 45-year-old female from Kumbakonam says, “It better to come here itself… It’s a mental satisfaction on coming and seeing them here.”
Theme 3: Suggestions	
Separate counter for PT-INR test	Participants say, “It would be good if we have a separate counter for our blood test.” A 48-year-old female says, “They(Doctors) see us after 11’ AM OPD. So if they separately see us at the same time, it would be better. It is also difficult to get buses back to our place during the night.”
Better seating facilities	A 40-year-old female says, “There is no place to sit. Also since they allow other test like urine test along with this, it take longer time. I have stood in the queue from the place where that AC runs.”

**Figure 1:**
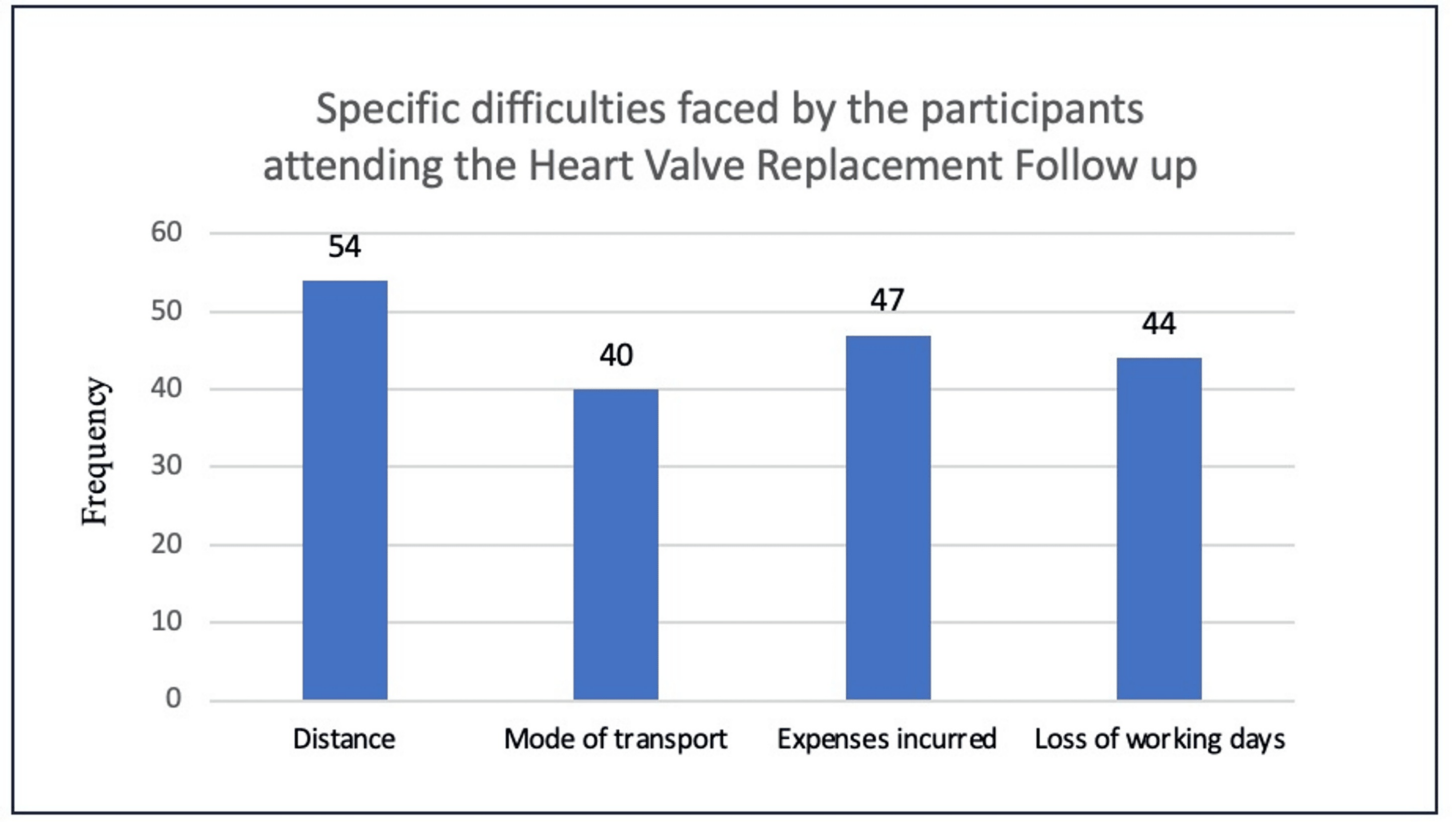
The proportion of participants facing specific difficulties in attending the monthly follow-up visits at the Heart Valve Replacement Clinic at a tertiary care center in Puducherry, India.

Travel-related difficulties include the unavailability of buses or direct buses, health issues related to travelling such as back pain, tiredness, huge crowds in the buses, etc. A large proportion of the people often have to leave the previous night itself and travel the whole night for the follow-up visit. At the hospital, they face difficulties like long waiting hours and the occasional unavailability of medicines and laboratory tests. They also face difficulties in the family like leaving the elderly alone at home, not being able to take care of educational matters of their children and dependence on others to take care of people at home (Figure 1).

## Discussion

Valvular heart disease is one of the most prevalent cardiac surgical conditions in India and often requires valve replacement surgery [1]. This underscores the need for patients to be on regular lifelong follow-up post-surgery [5]. In such cases, the expenses and difficulties significantly impact the patients. In our study, the median OOPE for a single monthly follow-up visit was Rs. 765, which is higher than the OOP OPD expenditure reported in other studies in India [13].

It was also noted that the total out-of-pocket (OOP) expenditure was primarily attributed to direct non-medical costs, while direct medical costs were minimal. The only direct medical expenditure incurred was for drugs unavailable at the hospital pharmacy. This was due to the study being conducted in a government setup where outpatient services, laboratory services, and certain medications were provided free of charge, thus significantly reducing direct medical expenditure. As shown in Table 2, indirect expenditure was mainly attributed to transportation costs. Thus, distance from the clinic was a major determinant of OOPE. The total OOPE increased with greater distance and in the presence of an accompanying person. As a result, patients were often compelled to attend follow-up visits alone to reduce costs.

Patients were found to face difficulties related to finance, transport, hospital, and family due to monthly follow-up visits. In the quantitative study, since patients registered only for outpatient department (OPD) attendance during follow-up, they were not covered under any insurance schemes such as *Ayushman Bharat*, which only covers the expenses of admitted patients [14]. This results in high recurring costs for patients, creating a significant financial burden. Quantitative analysis indicates that financial burden is one of the main issues they face (33%) due to follow-up visits. Patients are often forced to take out loans and may even compromise on basic needs such as food. The situation is further worsened by the fact that patients lose wages for a day or more when attending follow-up visits. Universal health coverage schemes primarily focus on preventive care and provide less attention to follow-up care, which compromises the quality of life for patients with chronic conditions.

Improved transportation enhances accessibility and thereby provides better healthcare to individuals [15]. The qualitative analysis revealed that people face difficulties related to travel, such as long travel hours, health issues related to travel, increased bus fares, and unavailability of buses. Transportation costs are also a significant contributor to the total OOPE. Thus, improving transport facilities or decreasing the distance of health facilities will help increase access and decrease the financial burden among the patients. The best way to address this issue is to decentralize follow-up care from tertiary care centers to lower levels using telemedicine. This approach would also help reduce the patient load at tertiary care centers. The majority of participants (73%) were willing to use telemedicine for follow-up care.

Patients also face difficulties at the hospital during follow-up visits, including long waiting queues, occasional unavailability of laboratory and other services, and difficulty accessing free medicines at the hospital pharmacy due to these queues. The situation worsens when a repeat follow-up visit within the same month is required due to deranged PT-INR values. These issues could be mitigated to some extent through the use of telemedicine follow-up, which would reduce the patient load at tertiary care centers and ease the burden on patients. Additionally, patients face difficulties in their family and personal lives. According to a study conducted by Whitehead et al., families play a crucial role in supporting and managing chronic illnesses [16]. Patients face family-related and personal difficulties, such as leaving elderly family members or children alone at home, depending on others to manage these issues, and disrupting their daily activities. They also struggle with taking care of children and missing school routines, such as parent meetings. All these also can be overcome by decreasing the follow-up timing by decentralizing follow-up care to nearby smaller hospitals. This can also be achieved through the use of telemedicine, as it helps decrease follow-up time [11]. Out of the total 143 participants in the study, approximately 43 (30%) were found to have irregular follow-up visits. This rate is higher than the 24% reported in similar studies conducted by Wray et al. [17].

The irregularity in follow-up visits was reported to be mainly due to work-related problems, financial constraints, other health issues, or a sense of well-being. It was also found that this irregularity was statistically associated with the period after surgery (*P*-value = 0.013). This implies that the regularity of follow-up visits decreases over time. It was observed that patients gradually become irregular after long periods of stable PT-INR values and learn to identify variations in their PT-INR values themselves, which explains the association.

The strength of the study lies in its mixed methods design, which provides a comprehensive understanding of the difficulties faced by patients. Additionally, the study includes an adequate sample size.

The expenditure for such patients visiting the OPD should also be covered under insurance schemes like Ayushman Bharat. Decentralizing follow-up care from tertiary care centers to lower levels using telemedicine can significantly reduce patient burdens by decreasing the patient load, easing access, and improving compliance. The technology of telemedicine must be employed for this process. Proper orientation and training should be provided to both doctors and patients regarding telemedicine follow-up to address and eliminate any concerns among patients. With the advent of COVID-19, digital health technologies have improved, potentially enhancing follow-up care at the primary level. New initiatives like Digisahayam, an assisted telemedicine solution developed by the Public Health Foundation of India (PHFI), aim to improve access to quality healthcare through bridge personnel trained to provide care. Such initiatives could be considered for involvement in follow-up care [18].

A limitation of the study is that, since it was conducted among patients attending the weekly valve replacement clinic, there is a higher chance of missing irregular patients who do not attend the clinic. The inclusion of only those actively attending follow-ups could lead to an underestimation of the OOPEs incurred by patients. Nonattenders may face higher travel or indirect costs due to difficulties in managing their conditions without professional guidance, which could result in increased emergency visits or hospitalizations that are not reflected in the collected data. In an attempt to reduce this bias, efforts were made to ensure representation from a range of socioeconomic backgrounds and demographics. Additionally, there is a higher chance of recall bias in the study, as the questionnaire relies on information provided by the patients. To minimize recall bias, patients were asked to recall information only about their most recent follow-up visit. The difficulties identified in this study should be considered and addressed.

## Conclusions

The qualitative study on the difficulties faced by patients due to follow-up visits revealed that patients encountered challenges related to financial issues, work, hospital experiences, family matters, and personal concerns. The proportion of patients with irregular follow-up visits was found to be 30.1%. The study highlights the significant financial burden faced by patients attending follow-up visits to a heart valve clinic post-surgery. The findings highlight the need for interventions to alleviate the financial strain on patients and their families. Policy measures could include subsidizing treatment costs, improving access to healthcare facilities, and providing financial assistance for travel and accommodation. Additionally, healthcare providers should offer counseling and support services to address the nonfinancial challenges faced by patients, ultimately enhancing the overall quality of care and patient outcomes in this population. Further research to evaluate the feasibility of tools like telemedicine for reducing difficulties in follow-up care would be valuable in easing the follow-up process. Research could focus on developing and evaluating support services, such as patient navigation programs, financial counseling, or community-based interventions, designed to alleviate some of the identified barriers.
